# Construction of a Family of Maximally Entangled Bases in **ℂ*^d^*** **⊗** **ℂ*^d′^***

**DOI:** 10.3390/e24030373

**Published:** 2022-03-06

**Authors:** Chenghong Wang, Kun Wang, Zhu-Jun Zheng

**Affiliations:** 1 School of Mathematics, South China University of Technology, Guangzhou 510641, China; machenghongwang2020@mail.scut.edu.cn; 2 College of Mathematics and Statistics, Zhoukou Normal University, Zhoukou 466001, China; wangkun@zknu.edu.cn; 3 Laboratory of Quantum Science and Engineering, South China University of Technology, Guangzhou 510641, China

**Keywords:** maximally entangled states, mutually unbiased bases, unextendible maximally entangled basis

## Abstract

In this paper, we present a new method for the construction of maximally entangled states in $C^{d}⊗C^{d^{\prime }}$ when $d^{\prime }\geq 2d$. A systematic way of constructing a set of maximally entangled bases (MEBs) in $C^{d}⊗C^{d^{\prime }}$ was established. Both cases when $d^{\prime }$ is divisible by *d* and not divisible by *d* are discussed. We give two examples of maximally entangled bases in $C^{2}⊗C^{4}$, which are mutually unbiased bases. Finally, we found a new example of an unextendible maximally entangled basis (UMEB) in $C^{2}⊗C^{5}$.

## 1. Introduction

Quantum entanglement is the phenomenon of particles interacting in a system consisting of two or more particles, although the particles may be separated by a distant space [1]. It is an important physical resource and plays an important role in quantum information processing, such as quantum computation [2], cryptographic protocols [3,4], quantum state tomography [5,6], and modern quantum technologies [7,8]. Specifically, the maximally entangled states play a central role in quantum mechanics and quantum information processing [9,10].

In 1960, mutually unbiased bases (MUBs), first introduced by Schwinger in [11], also have useful applications in quantum information processing, and Ivanovic applied the mutually unbiased bases to the problem of quantum state determination in [12]. Two orthonormal bases $B_{1}=\{|\phi_{i}\rangle {\}}_{i\;=\;0}^{dd^{\prime }-1}$ and $B_{2}=\{|\psi_{j}\rangle {\}}_{j\;=\;0}^{dd^{\prime }-1}$ of $C^{d}⊗C^{d^{\prime }}$ are called mutually unbiased if and only if:

$$\left|\langle \phi_{i}|\psi_{j}\rangle \right|=\frac{1}{\sqrt{dd^{\prime }}},\mathrm{for}\;\mathrm{any}\;i,j=0,1,\ldots ,dd^{\prime }.$$

A set of orthonormal bases $\left\{B_{1},B_{2},\ldots ,B_{k}\right\}$ is called mutually unbiased if any two bases of them are mutually unbiased. Denote N(d) the maximum number of any set of MUBs in $C^{d}$. An open problem with mutually unbiased bases is to determine the value of N(d) when *d* is not a power of a prime number. It is already known that N(d)≤d+1 for any dimension *d* and N(d)=d+1 if *d* is a prime power [13,14,15]. However, if *d* has at least two different prime divisors, the result for N(d) is known very little, even for d=6. We refer the readers to [16] and the references cited in that paper.

The unextendible maximally entangled basis (UMEB) in $C^{d}⊗C^{d}$ was introduced in [17]. It was shown that the UMEB is constructed explicitly when d=3 and d=4[18]. In Reference [18], Chen and Fei studied the UMEB in any bipartite system $C^{d}⊗C^{d^{\prime }}$. They presented a method to construct $d^{2}$-member UMEBs in $C^{d}⊗C^{d^{\prime }}$ when $d^{\prime }<2d<2d^{\prime }$ and gave two examples of mutually unbiased unextendible maximally entangled bases (MUUMEBs) in $C^{2}⊗C^{3}$. In recent years, UMEBs have been studied more extensively in arbitrary bipartite systems. In [19], Nan et al. presented the construction of the $qd^{2}$-member UMEBs in $C^{d}⊗C^{d^{\prime }}$ when $d^{\prime }=qd+r,0<r<d$ and gave two complete MUUMEBs in $C^{3}⊗C^{4}$. In [20], Tao et al. studied the mutually unbiased maximally entangled bases in bipartite systems $C^{d}⊗C^{kd}$. They presented five MEBs in $C^{2}⊗C^{4}$ and three MEBs in $C^{2}⊗C^{6}$ that are mutually unbiased. In [21], Zhang et al. provided two constructions of UMEBs in $C^{pd}⊗C^{qd}$ based on the constructions of UMEBs in $C^{d}⊗C^{d}$ and in $C^{p}⊗C^{q}$. In [22], Han et al. presented an easy way of constructing a mutually unbiased entangled basis with a fixed Schmidt number of two (MUSEB2) in $C^{3}⊗C^{4k}$ when 3∤k. In [23], Tang et al. constructed two complete UMEBs in bipartite system $C^{d}⊗C^{q(d+1)}$ and obtained the sufficient and necessary conditions of these two UMEBs to develop MUBs. In [24], Xu constructed new types of mutually unbiased maximally entangled bases (MUMEBs) in $C^{2^{s}}⊗C^{2^{s}}$ by using Galois rings.

In this paper, we provide a new construction of MEBs in bipartite system $C^{d}⊗C^{d^{\prime }}$ when $d^{\prime }\geq 2d$. We first review some basic concepts, then present a systematic way of constructing MEBs, which is a different approach from [18,20] and Theorem 1 in [19]. To briefly summarize their methods, these three articles obtained a new set of MEBs by using all the operators of the Weyl–Heisenberg group of the low-dimensional system to act on the standard maximally entangled state. None of them applied the Weyl–Heisenberg group of the high-dimensional system to construct MEBs. In this paper, we shall consider this type of construction of MEBs. The difficult problem we need to overcome is choosing a part of the operators in the Weyl–Heisenberg group and finding a suitable state. Since the operators of the Weyl–Heisenberg group are invertible, the MEBs we construct are certainly different from those of [18,19,20]. Furthermore, we give explicit examples of MEBs in $C^{2}⊗C^{4}$, which are mutually unbiased, and eight-member UMEBs in $C^{2}⊗C^{5}$. Finally, we give some conclusions and discussions of this paper.

## 2. Preliminaries

For the sake of convenience, we review some basic definitions and notations for quantum entanglement states in the following.

Suppose the Hilbert space associated with some isolated physical bipartite system is $H_{A}⊗H_{B}=C^{d}⊗C^{d^{\prime }}$. Let {|0〉,|1〉,…,|d−1〉} and $\{|0^{\prime }\rangle ,|1^{\prime }\rangle ,\ldots ,|{\left(d^{\prime }-1\right)}^{\prime }\rangle \}$ be the computational bases in $C^{d}$ and $C^{d^{\prime }}$, respectively. A state |ϕ〉 in $C^{d}⊗C^{d^{\prime }}$ is called a product state (or separable state) if it can be written as $\left|\phi \rangle =\right|\phi_{1}\rangle ⊗|\phi_{2}\rangle$, where $|\phi_{1}\rangle \in C^{d}$ and $|\phi_{2}\rangle \in C^{d^{\prime }}$ are any two quantum states of the corresponding subsystems. Otherwise, the state |ϕ〉 is called an entangled state. For any given orthonormal complete basis $\{|a_{i}\rangle {\}}_{i=0}^{d-1}$ of subsystem *A*, if there exists an orthonormal basis $\{|b_{j}\rangle {\}}_{j=0}^{d^{\prime }-1}$ of subsystem *B* such that |ϕ〉 can be expressed as $|\phi \rangle =\frac{1}{\sqrt{d}}\sum_{i=0}^{d-1}|a_{i}\rangle ⊗|b_{i}\rangle$, then |ϕ〉 is said to be a maximally entangled state [18].

One can also describe the maximally entangled state by the so-called Schmidt decomposition. For any vector $|\psi \rangle \in C^{d}⊗C^{d^{\prime }}$, one has the corresponding Schmidt decomposition [25]:

(1) $$|\psi \rangle =\sum_{k=1}^{r}\lambda_{k}|c_{k}\rangle ⊗|e_{k}\rangle ,$$

where $\lambda_{k}\left(1\leq k\leq r\right)$ are positive real numbers and $\left\{c_{i}\right\}$, $\left\{e_{j}\right\}$ are orthonormal bases in $C^{d}$ and $C^{d^{\prime }}$, respectively. If |ψ〉 is a pure state of a composite system, then $\lambda_{k}\left(1\leq k\leq r\right)$ are called its Schmidt coefficients and r=Sr(|ψ〉) the Schmidt number. The pure state |ψ〉 is maximally entangled if and only if its Schmidt number is *d* and all Schmidt coefficients are equal to $\frac{1}{\sqrt{d}}$.

We denote by $M_{d^{\prime }\times d}\left(C\right)$ the vector space of all $d^{\prime }\times d$ complex matrices. $M_{d^{\prime }\times d}\left(C\right)$ is a Hilbert space under the Hilbert–Schmidt inner product defined by $\langle X,Y\rangle \equiv \mathrm{Tr}\left(X^{†}Y\right)$ for any two $d^{\prime }\times d$ matrices X,Y. If $|\phi \rangle =\frac{1}{\sqrt{d}}\sum_{i=0}^{d-1}\sum_{j=0}^{d^{\prime }-1}x_{ji}\left|i\rangle \right|j^{\prime }\rangle$ is a pure state in $C^{d}⊗C^{d^{\prime }}$, then there is a corresponding $d^{\prime }\times d$ matrix $M=M(|\phi \rangle )=\left(x_{ji}\right)$, and the Schmidt number of |ϕ〉 and the rank of matrix *M* are equal. Moreover, if |ϕ〉 and |ψ〉 are two pure states in $C^{d}⊗C^{d^{\prime }}$, then 〈M(|ϕ〉),M(|ψ〉)〉=d〈ϕ|ψ〉 [26,27]. It is easy to see that |ϕ〉 is a maximally entangled state if and only if all the singular values of the matrix M(|ϕ〉) equal one.

A collection of states $\{|\phi_{i}\rangle \in C^{d}⊗C^{d^{\prime }}|1\leq i\leq n,n<dd^{\prime }\}$ is called an unextendible maximally entangled basis (UMEB) [18] if and only if:

**(i)**  $|\phi_{i}\rangle \left(1\leq i\leq n\right)$ are all maximally entangled states;
**(ii)**  $\langle \phi_{i}|\phi_{j}\rangle =\delta_{ij}$, for 1≤i,j≤n;
**(iii)**  All the states in the orthogonal complement space of $\mathrm{span}\{|\phi_{i}\rangle {\}}_{i=1}^{n}$ cannot be maximally entangled.

Next, let us consider a set of d×d unitary matrices:

(2) $$U_{n,m}=\sum_{k=0}^{d-1}\alpha_{d}^{kn}|k⊕m\rangle \langle k|,$$

where n,m=0,1,…,d−1, $\alpha_{d}$ is any primitive *d*th root of unity, and k⊕m denotes (k+m)modd. These $d^{2}$ matrices constitute a basis of the vector space $M_{d\times d}\left(C\right)$ (or equivalently, the operator space on $C^{d}$) and:

(3) $$\langle U_{n_{1},m_{1}},U_{n_{2},m_{2}}\rangle =\mathrm{Tr}\left(U_{n_{1},m_{1}}^{†}U_{n_{2},m_{2}}\right)=d\delta_{n_{1},n_{2}}\delta_{m_{1},m_{2}}.$$

The above $d^{2}$ linear transformations $U_{n,m}$ correspond to the Weyl–Heisenberg group. We use some of these operators to construct maximally entangled bases in $C^{d}⊗C^{d^{\prime }}$, which is a different method compared to that in [18,20].

## 3. MEBs in ℂ*^d^* ⊗ ℂ*^qd^*

In this section, we present a new method of constructing a maximally entangled basis (MEB) in $C^{d}⊗C^{qd}$ when q≥2.

Consider the following pure state:

(4) $$|\phi_{d}^{\left(q\right)}\rangle =\frac{1}{\sqrt{qd}}\sum_{k=0}^{d-1}\left[|k\rangle ⊗(|{\left(qk\right)}^{\prime }\rangle {+|\left(qk+1\right)}^{\prime }{\rangle +\cdots +|\left(qk+q-1\right)}^{\prime }\rangle )\right].$$

If we assume q=1, then the state in (4) happens to be the standard maximally entangled state in $C^{d}⊗C^{d}$. In [18,19,20], they all used this type of state to construct mutually unbiased MEBs and unextendible MEBs by making a transformation to the bases of subsystems. In this case, our constructions coincide with theirs. However, when q≥2, we did obtain a different kind of maximally entangled states. In our case, it is not difficult to check that all the singular values of the complex matrix $M(|\phi_{d}^{\left(q\right)}\rangle )$ (the definition of this complex matrix is in the Section 2) equal one. That is to say, $|\phi_{d}^{\left(q\right)}\rangle$ is a maximally entangled state.

Now, let $U_{n,m},n,m=0,1,\ldots ,qd-1$ be the $q^{2}d^{2}$ operators on $C^{qd}$. We apply these operators on the above state as follows:

(5) $$|\phi_{n,m}^{\left(q\right)}\rangle =\left(I_{d}⊗U_{n,m}\right)|\phi_{d}^{\left(q\right)}\rangle .$$

Obviously, we obtain $q^{2}d^{2}$ maximally entangled states in $C^{d}⊗C^{qd}$. These states cannot form a basis of $C^{d}⊗C^{qd}$ since $q^{2}d^{2}>qd^{2}$. However, we can always choose part of the states in (5). Next, we briefly list some simple properties of the Weyl–Heisenberg group. Based on this, we found a family of maximally entangled bases of form (5). Then, in particular, we illustrate our method with two examples of low-dimensional systems.

We first considered the $d^{2}$ unitary matrices $U_{n,m},n,m=0,1,\ldots ,d-1$ defined in (2) of the vector space $M_{d\times d}\left(C\right)$. Each of these $d^{2}$ matrices has only *d* nonzero entries. These nonzero entries are all the *d*th root of unity. The form of these matrices $U_{n,m}$ is determined by *m*, which is independent of the value of *n*. When m=0, $U_{n,0}$ is a diagonal matrix, and the entries on the diagonal are the *d*th root of unity. When m=1, the nonzero entry of the first row is in the last column, and the other d−1 nonzero entries are directly below the main diagonal, i.e., they are in the lower left subdiagonal of the matrix. When m=d−1, the *d* entries of the bottom left corner and the superdiagonal of the matrix are nonzero elements. The nonzero entries of these matrices move parallel along the main diagonal. There are exactly *d* different forms of matrices. There are exactly *d* matrices in each form, which are determined by the value of *n*. For example, we have *d* diagonal matrices in the Weyl–Heisenberg group. According to group theory, the form of the product of two matrices of the Weyl–Heisenberg group is also one of these *d* different types.

Now, let us discuss the maximally entangled states defined in (5). Let $n,m,n^{\prime },m^{\prime }\in \left\{0,1,\ldots ,qd-1\right\}$ and $U_{n,m},U_{n^{\prime },m^{\prime }}$ be two operators on $C^{qd}$. Denote the matrix $U_{n^{\prime },m^{\prime }}^{†}U_{n,m}$ by ${\left(u_{ij}\right)}_{0\leq i,j\leq qd-1}$. If $\left(n,m\right)=\left(n^{\prime },m^{\prime }\right)$, then by (3), we have:

$$\langle \phi_{n^{\prime },m^{\prime }}^{\left(q\right)}|\phi_{n,m}^{\left(q\right)}\rangle =\langle \phi_{d}^{\left(q\right)}|\left(I_{d}⊗U_{n^{\prime },m^{\prime }}^{†}U_{n,m}\right)|\phi_{d}^{\left(q\right)}\rangle =1.$$

If $\left(n,m\right)\neq \left(n^{\prime },m^{\prime }\right)$, after a simple calculation, we have:

$$\begin{array}{c} \langle \phi_{n^{\prime },m^{\prime }}^{\left(q\right)}|\phi_{n,m}^{\left(q\right)}\rangle =u_{01}+u_{02}+\cdots +u_{0,q-1}\\ \;\;\;\;\;\;\;\;\;\;\;\;\;\;\;\;\;\;\;\;\;+u_{10}+u_{12}+\cdots +u_{1,q-1}\\ \;\;\;\;\;\;\;\;\;\;\;\;\;\;\;\;\;\;\;\;\;+\cdots \\ \;\;\;\;\;\;\;\;\;\;\;\;\;\;\;\;\;\;\;\;\;+u_{q-1,0}+u_{q-1,1}+\cdots +u_{q-1,q-2}\\ \;\;\;\;\;\;\;\;\;\;\;\;\;\;\;\;\;\;\;\;\;+u_{q,q+1}+u_{q,q+2}+\cdots +u_{q,2q-1}\\ \;\;\;\;\;\;\;\;\;\;\;\;\;\;\;\;\;\;\;\;\;+u_{q+1,q}+u_{q+1,q+2}+\cdots +u_{q+1,2q-1}\\ \;\;\;\;\;\;\;\;\;\;\;\;\;\;\;\;\;\;\;\;\;+\cdots \\ \;\;\;\;\;\;\;\;\;\;\;\;\;\;\;\;\;\;\;\;\;+u_{2q-1,q}+u_{2q-1,q+1}+\cdots +u_{2q-1,2q-2}\\ \;\;\;\;\;\;\;\;\;\;\;\;\;\;\;\;\;\;\;\;\;+\cdots \\ \;\;\;\;\;\;\;\;\;\;\;\;\;\;\;\;\;\;\;\;\;+u_{q(d-1),q(d-1)+1}+u_{q(d-1),q(d-1)+2}+\cdots +u_{q(d-1),qd-1}\\ \;\;\;\;\;\;\;\;\;\;\;\;\;\;\;\;\;\;\;\;\;+u_{q(d-1)+1,q(d-1)}+u_{q(d-1)+1,q(d-1)+2}+\cdots +u_{q(d-1)+1,qd-1}\\ \;\;\;\;\;\;\;\;\;\;\;\;\;\;\;\;\;\;\;\;\;+\cdots \\ \;\;\;\;\;\;\;\;\;\;\;\;\;\;\;\;\;\;\;\;\;+u_{qd-1,q(d-1)}+u_{qd-1,q(d-1)+1}+\cdots +u_{qd-1,qd-2}. \end{array}$$

In order to make the right-hand side of the above formula equal to zero, we need to find out how many forms of the matrix in the Weyl–Heisenberg group satisfy that the corresponding elements are all zero. Since $u_{0,q-1},u_{q,2q-1},\cdots ,u_{q(d-1),qd-1}$ are parallel to the main diagonal and all the other $u_{ij}$’s appearing in the above formula are closer to the main diagonal, we have q−1 forms of matrices in the Weyl–Heisenberg that make these entries not equal to zero. According to our analysis in the previous paragraph, if we choose *m* or $m^{\prime }$ from the set {0,q,2q,⋯,(d−1)q}, then the sum of the $u_{ij}$’s of the right-hand side of the above equation is equal to zero. Because there are qd matrices in each form (that is, *n* can be chosen from all these numbers {0,1,2,⋯,qd−1}), thus we find $qd\times d=qd^{2}$ matrices to make $|\phi_{n,m}^{\left(q\right)}\rangle$ satisfy the orthogonal property. At this point, we have proven that we obtained a set of maximally entangled bases. In order to make our construction clearer, we illustrate this method with two examples below by writing the states in detail.

Let us first construct MEBs in $C^{2}⊗C^{6}\left(d=2,q=3\right)$. According to the above construction, we have:

(6) $$|\phi_{2}^{\left(3\right)}\rangle =\frac{1}{\sqrt{6}}\left[|0\rangle ⊗(|0^{\prime }\rangle +|1^{\prime }\rangle +|2^{\prime }\rangle )+|1\rangle ⊗(|3^{\prime }\rangle +|4^{\prime }\rangle +|5^{\prime }\rangle )\right].$$

We chose n=0,1,2,3,4,5 and m=0,3, such that:

(7) $$|\phi_{n,m}^{\left(3\right)}\rangle =\left(I_{2}⊗U_{n,m}\right)|\phi_{2}^{\left(3\right)}\rangle .$$

Since:

(8) $$\begin{array}{cc} \; & \langle \phi_{n^{\prime },m^{\prime }}^{\left(3\right)}|\phi_{n,m}^{\left(3\right)}\rangle =\frac{1}{6}\mathrm{Tr}\left(U_{n^{\prime },m^{\prime }}^{†}U_{n,m}\right)+u_{01}+u_{12}+u_{10}+u_{21}\\ \; & \;\;\;\;\;\;\;\;\;\;\;\;\;\;\;\;\;\;\;\;\;\;\;\;+u_{34}+u_{43}+u_{45}+u_{54}+u_{02}+u_{20}+u_{35}+u_{53}, \end{array}$$

where we denote the matrix $U_{n^{\prime },m^{\prime }}^{†}U_{n,m}={\left(u_{ij}\right)}_{0\leq i,j\leq 5}$. We derived that the sum of $u_{ij}$’s in Equation (8) is equal to zero when m=0,3. It is easy now to check that the above 12 states exactly form an orthonormal maximally entangled basis in $C^{2}⊗C^{6}$.

Next, we deal with the maximally entangled bases in $C^{3}⊗C^{6}\left(d=3,q=2\right)$. Similarly, we have:

(9) $$|\phi_{3}^{\left(2\right)}\rangle =\frac{1}{\sqrt{6}}\left[|0\rangle ⊗(|0^{\prime }\rangle +|1^{\prime }\rangle )+|1\rangle ⊗(|2^{\prime }\rangle +|3^{\prime }\rangle )+|2\rangle ⊗(|4^{\prime }\rangle +|5^{\prime }\rangle )\right].$$

In this case, we chose n=0,1,2,3,4,5 and m=0,2,4, such that:

(10) $$|\phi_{n,m}^{\left(2\right)}\rangle =\left(I_{2}⊗U_{n,m}\right)|\phi_{3}^{\left(2\right)}\rangle .$$

Since:

(11) $$\langle \phi_{n^{\prime },m^{\prime }}^{\left(2\right)}|\phi_{n,m}^{\left(2\right)}\rangle =\frac{1}{6}\mathrm{Tr}\left(U_{n^{\prime },m^{\prime }}^{†}U_{n,m}\right)+u_{01}+u_{10}+u_{23}+u_{32}+u_{45}+u_{54},$$

where we denote again the matrix $U_{n^{\prime },m^{\prime }}^{†}U_{n,m}={\left(u_{ij}\right)}_{0\leq i,j\leq 5}$. Similar to the previous case, the 18 states constitute an orthonormal maximally entangled basis in $C^{3}⊗C^{6}$.

## 4. MUMEBs in ℂ^2^ ⊗ ℂ^4^

In this section, we shall study the mutually unbiased maximally entangled bases (MUMEBs) in $C^{2}⊗C^{4}$. Let {|0〉,|1〉} and $\{|0^{\prime }\rangle ,|1^{\prime }\rangle ,|2^{\prime }\rangle ,|3^{\prime }\rangle \}$ be the computational bases in $C^{2}$ and $C^{4}$, respectively. Throughout this section, $\left\{U_{n,m}|n,m=0,1,2,3\right\}$ defined in the Section 2 is the set of 16 operators on $C^{4}$.

Let:

(12) $$|\phi_{2}^{\left(2\right)}\rangle =\frac{1}{2}\left[|0\rangle ⊗(|0^{\prime }\rangle +|1^{\prime }\rangle )+|1\rangle ⊗(|2^{\prime }\rangle +|3^{\prime }\rangle )\right]$$

and:

$$\begin{array}{cc} \; & |\phi_{n,m}^{\left(2\right)}\rangle \\ \; & =\left(I_{2}⊗U_{n,m}\right)|\phi^{\left(2\right)}\rangle \\ \; & =\frac{1}{2}\left[|0\rangle ⊗U_{n,m}(|0^{\prime }\rangle +|1^{\prime }\rangle )+|1\rangle ⊗U_{n,m}(|2^{\prime }\rangle +|3^{\prime }\rangle )\right]\\ \; & =\frac{1}{2}\left[|0\rangle (|m^{\prime }\rangle +i^{n}|{\left(m⊕1\right)}^{\prime }\rangle )+|1\rangle (i^{2n}|{\left(m⊕2\right)}^{\prime }\rangle +i^{3n}|{\left(m⊕3\right)}^{\prime }\rangle )\right], \end{array}$$

where m⊕j denotes (m+j)mod4 for j=1,2,3.

In this case, we have d=2 and q=2. Thus, the value of *m* = 0,2. According to the previous discussion of our construction of the maximally entangled basis and after a simple calculation, we obtained that:

(13) $$\left\{|\phi_{n,m}^{\left(2\right)}\rangle |n=0,1,2,3;m=0,2\right\}$$

is a maximally entangled basis in $C^{2}⊗C^{4}$.

Suppose $\{|b_{0}\rangle ,|b_{1}\rangle \}$ is another basis in $C^{2}$ and the transition matrix from {|0〉,|1〉} to $\{|b_{0}\rangle ,|b_{1}\rangle \}$ is the Hadamard matrix:

(14) $$\frac{1}{\sqrt{2}}\left(\begin{array}{cc} 1 & 1\\ 1 & -1 \end{array}\right).$$

Then we have

(15) $$\left(\begin{array}{c} |b_{0}\rangle \\ |b_{1}\rangle \end{array}\right)=\frac{1}{\sqrt{2}}\left(\begin{array}{cc} 1 & 1\\ 1 & -1 \end{array}\right)\left(\begin{array}{c} |0\rangle \\ |1\rangle \end{array}\right).$$

After a simple calculation, we obtain another maximally entangled basis in $C^{2}⊗C^{4}$:

(16) $$|\psi {{}^{\prime }}_{n,m}^{\left(2\right)}\rangle =\frac{1}{2}\left[|b_{0}\rangle ⊗U_{n,m}(|0^{\prime }\rangle +|1^{\prime }\rangle )+|b_{1}\rangle ⊗U_{n,m}(|2^{\prime }\rangle +|3^{\prime }\rangle )\right],$$

where n=0,1,2,3;m=0,2.

It is easy to check that the bases $\left\{|\psi {{}^{\prime }}_{n,m}^{\left(2\right)}\rangle \right\}$ and $\left\{|\phi_{n,m}^{\left(2\right)}\rangle \right\}$ in $C^{2}⊗C^{4}$ are not mutually unbiased. In order to obtain a mutually unbiased basis, we just need to match the above states with some coefficients. That is, let:

$$\begin{array}{cc} \; & |\psi_{n,m}^{\left(2\right)}\rangle \\ \; & =\frac{1}{2}\left[|b_{0}\rangle ⊗U_{n,m}(x_{0}|0^{\prime }\rangle +x_{1}|1^{\prime }\rangle )+|b_{1}\rangle ⊗U_{n,m}(x_{2}|2^{\prime }\rangle +x_{3}|3^{\prime }\rangle )\right]\\ \; & =\frac{1}{2}\left[\right|b_{0}\rangle ⊗(x_{0}|m^{\prime }\rangle +i^{n}x_{1}|{\left(m⊕1\right)}^{\prime }\rangle )\\ \; & \;\;\;\;\;\;\;\;\;+|b_{1}\rangle ⊗(i^{2n}x_{2}|{\left(m⊕2\right)}^{\prime }\rangle +i^{3n}x_{3}{|\left(m⊕3\right)}^{\prime }\rangle )], \end{array}$$

where n=0,1,2,3;m=0,2. As for the coefficients, we have the following four inequivalent choices:

(17) $$\left(\begin{array}{c} x_{0}\\ x_{1}\\ x_{2}\\ x_{3} \end{array}\right)=\left(\begin{array}{c} 1\\ 1\\ 1\\ -1 \end{array}\right),\left(\begin{array}{c} 1\\ -1\\ 1\\ 1 \end{array}\right),\left(\begin{array}{c} 1\\ i\\ -1\\ i \end{array}\right)\mathrm{or}\left(\begin{array}{c} 1\\ -i\\ -1\\ -i \end{array}\right).$$

Now, it is not difficult to prove that the basis $B_{1}=\{|\phi_{n,m}^{\left(2\right)}\rangle {\}}_{n\;=\;0,1,2,3}^{m\;=\;0,2}$ and the basis $B_{2}=\{|\psi_{s,t}^{\left(2\right)}\rangle {\}}_{s\;=\;0,1,2,3}^{t\;=\;0,2}$ are mutually unbiased. In other words, we have:

(18) $$\left|\langle \phi_{n,m}^{\left(2\right)}|\psi_{s,t}^{\left(2\right)}\rangle \right|=\frac{1}{2\sqrt{2}},\mathrm{for}\;\mathrm{any}\;n,m,s,t.$$

Let us choose another basis $\{|c_{0}\rangle ,|c_{1}\rangle \}$ in $C^{2}$ as:

(19) $$\left(\begin{array}{c} |c_{0}\rangle \\ |c_{1}\rangle \end{array}\right)=\frac{1}{\sqrt{2}}\left(\begin{array}{cc} 1 & \sqrt{-1}\\ \sqrt{-1} & 1 \end{array}\right)\left(\begin{array}{c} |0\rangle \\ |1\rangle \end{array}\right).$$

Thus, we obtain the following maximally entangled states:

$$\begin{array}{cc} \; & |\gamma_{n,m}^{\left(2\right)}\rangle \\ \; & =\frac{1}{2}\left[|c_{0}\rangle ⊗U_{n,m}(y_{0}|0^{\prime }\rangle +y_{1}|1^{\prime }\rangle )+|c_{1}\rangle ⊗U_{n,m}(y_{2}|2^{\prime }\rangle +y_{3}|3^{\prime }\rangle )\right]\\ \; & =\frac{1}{2}\left[\right|c_{0}\rangle ⊗(y_{0}|m^{\prime }\rangle +i^{n}y_{1}|{\left(m⊕1\right)}^{\prime }\rangle )\\ \; & \;\;\;\;\;\;\;\;\;+|c_{1}\rangle ⊗(i^{2n}y_{2}|{\left(m⊕2\right)}^{\prime }\rangle +i^{3n}y_{3}{|\left(m⊕3\right)}^{\prime }\rangle )], \end{array}$$

where n=0,1,2,3;m=0,2. If we let the coefficients $\left\{y_{0},y_{1},y_{2},y_{3}\right\}$ be any four of the following:

(20) $$\left(\begin{array}{c} y_{0}\\ y_{1}\\ y_{2}\\ y_{3} \end{array}\right)=\left(\begin{array}{c} 1\\ -1\\ -1\\ -1 \end{array}\right),\left(\begin{array}{c} 1\\ 1\\ -1\\ 1 \end{array}\right),\left(\begin{array}{c} 1\\ i\\ 1\\ -i \end{array}\right)\mathrm{or}\left(\begin{array}{c} 1\\ -i\\ 1\\ i \end{array}\right),$$

then we obtain that the basis $B_{1}=\{|\phi_{n,m}^{\left(2\right)}\rangle {\}}_{n=0,1,2,3}^{m=0,2}$ and the basis $B_{3}=\{|\gamma_{k,l}^{\left(2\right)}\rangle {\}}_{k\;=\;0,1,2,3}^{l\;=\;0,2}$ are mutually unbiased. That is to say,

(21) $$\left|\langle \phi_{n,m}^{\left(2\right)}|\gamma_{k,l}^{\left(2\right)}\rangle \right|=\frac{1}{2\sqrt{2}},\mathrm{for}\;\mathrm{any}\;n,m,k,l.$$

To summarize this section, we need to explain that $\left\{B_{1},B_{2},B_{3}\right\}$ is not a mutually unbiased maximally entangled basis in $C^{2}⊗C^{4}$. One can check this straightforwardly by the definition of mutually unbiased bases that $B_{2}$ and $B_{3}$ are not unbiased. We found that this is related to the selection of *m*. We hope that this will be helpful to the research of mutually unbiased bases.

## 5. UMEBs in ℂ^2^ ⊗ ℂ^5^

In this section, we discuss a new construction of unextendible maximally entangled bases (UMEBs) in $C^{2}⊗C^{5}$. Let {|0〉,|1〉} and $\{|0^{\prime }\rangle ,|1^{\prime }\rangle ,|2^{\prime }\rangle ,|3^{\prime }\rangle ,|4^{\prime }\rangle \}$ be the computational bases in $C^{2}$ and $C^{5}$, respectively. We prove the following maximally entangled states form of the UMEB in $C^{2}⊗C^{5}$:

(22) $$|\tau_{n,0}\rangle =\frac{1}{2}\left[|0\rangle (|0^{\prime }\rangle +i^{n}|1^{\prime }\rangle )+|1\rangle (i^{2n}|2^{\prime }\rangle +i^{3n}|3^{\prime }\rangle )\right],$$

(23) $$|\tau_{n,2}\rangle =\frac{1}{2}\left[|0\rangle (|2^{\prime }\rangle +i^{n}|3^{\prime }\rangle )+|1\rangle (i^{2n}|0^{\prime }\rangle +i^{3n}|1^{\prime }\rangle )\right],$$

where n=0,1,2,3.

We used reductio ad absurdum to prove the above statement. Suppose there exists an extended maximally entangled state:

(24) $$|\eta \rangle =\left(X⊗Y\right)(a_{0}|0\rangle ⊗|0^{\prime }\rangle +a_{1}\left|1\rangle ⊗\right|1^{\prime }\rangle ),$$

where $X={\left(x_{ij}\right)}_{2\times 2}$ and $Y={\left(y_{ij}\right)}_{5\times 5}$ are two unitary operators on $C^{2}$ and $C^{5}$ with respect to the above bases. Then, for all n=0,1,2,3, we have:

(25) $$\langle \tau_{n,0}|\eta \rangle =0,\langle \tau_{n,2}|\eta \rangle =0.$$

Let us compute these two equations in two steps.

At first, by a direct computation, we have:

$$\begin{array}{cc} \; & \langle \tau_{n,0}|\eta \rangle \\ = & \left[\langle 0|⊗\left(\langle 0\right|+\langle 1|i^{n})+\langle 1|⊗\left(\langle 2\right|i^{2n}+\langle 3|i^{3n})\right]\\ \; & \;\;\left[a_{0}(X|0\rangle )⊗(Y|0^{\prime }\rangle )+a_{1}(X|1\rangle )⊗(Y|1^{\prime }\rangle )\right]\\ = & a_{0}\langle 0|X|0\rangle \left[(\langle 0^{\prime }|+\langle 1^{\prime }|i^{n})Y|0^{\prime }\rangle \right]+a_{1}\langle 0|X|1\rangle \left[(\langle 0^{\prime }|+\langle 1^{\prime }|i^{n})Y|1^{\prime }\rangle \right]\\ \; & \;\;+a_{0}\langle 1|X|0\rangle \left[(\langle 2^{\prime }|i^{2n}+\langle 3^{\prime }|i^{3n})Y|0^{\prime }\rangle \right]+a_{1}\langle 1|X|1\rangle \left[(\langle 2^{\prime }|i^{2n}+\langle 3^{\prime }|i^{3n})Y|1^{\prime }\rangle \right]\\ = & \;a_{0}x_{11}\left(y_{11}+i^{n}y_{21}\right)+a_{1}x_{12}\left(y_{12}+i^{n}y_{22}\right)\\ \; & \;\;+a_{0}x_{21}\left(i^{2n}y_{31}+i^{3n}y_{41}\right)+a_{1}x_{22}\left(i^{2n}y_{32}+i^{3n}y_{42}\right)\\ = & \;0. \end{array}$$

Substitute n=0,1,2,3 into the above formula, we have the following four equations:

(26) $$\begin{array}{cc} \; & a_{0}x_{11}\left(y_{11}+y_{21}\right)+a_{1}x_{12}\left(y_{12}+y_{22}\right)\\ \; & \;\;\;\;\;\;+a_{0}x_{21}\left(y_{31}+y_{41}\right)+a_{1}x_{22}\left(y_{32}+y_{42}\right)=0, \end{array}$$

(27) $$\begin{array}{cc} \; & a_{0}x_{11}\left(y_{11}+iy_{21}\right)+a_{1}x_{12}\left(y_{12}+iy_{22}\right)\\ \; & \;\;\;\;\;\;-a_{0}x_{21}\left(y_{31}+iy_{41}\right)-a_{1}x_{22}\left(y_{32}+iy_{42}\right)=0, \end{array}$$

(28) $$\begin{array}{cc} \; & a_{0}x_{11}\left(y_{11}-y_{21}\right)+a_{1}x_{12}\left(y_{12}-y_{22}\right)\\ \; & \;\;\;\;\;\;+a_{0}x_{21}\left(y_{31}-y_{41}\right)+a_{1}x_{22}\left(y_{32}-y_{42}\right)=0, \end{array}$$

(29) $$\begin{array}{cc} \; & a_{0}x_{11}\left(y_{11}-iy_{21}\right)+a_{1}x_{12}\left(y_{12}-iy_{22}\right)\\ \; & \;\;\;\;\;\;-a_{0}x_{21}\left(y_{31}-iy_{41}\right)-a_{1}x_{22}\left(y_{32}-iy_{42}\right)=0. \end{array}$$

Combining the above equations, that is (26) ± (28) and (27) ± (29), respectively, after a simplification, we obtain the following four equivalent equations:

(30) $$\begin{array}{ccc} a_{0}x_{11}y_{11}+a_{1}x_{12}y_{12}+a_{0}x_{21}y_{31}+a_{1}x_{22}y_{32} & = & 0, \end{array}$$

(31) $$\begin{array}{ccc} a_{0}x_{11}y_{21}+a_{1}x_{12}y_{22}+a_{0}x_{21}y_{41}+a_{1}x_{22}y_{42} & = & 0, \end{array}$$

(32) $$\begin{array}{ccc} a_{0}x_{11}y_{11}+a_{1}x_{12}y_{12}-a_{0}x_{21}y_{31}-a_{1}x_{22}y_{32} & = & 0, \end{array}$$

(33) $$\begin{array}{ccc} a_{0}x_{11}y_{21}+a_{1}x_{12}y_{22}-a_{0}x_{21}y_{41}-a_{1}x_{22}y_{42} & = & 0. \end{array}$$

Secondly, according to $\langle \tau_{n,2}|\eta \rangle =0$, we have:

(34) $$\begin{array}{cc} \; & a_{0}x_{11}\left(y_{31}+y_{41}\right)+a_{1}x_{12}\left(y_{32}+y_{42}\right)\\ \; & \;\;\;\;\;\;+a_{0}x_{21}\left(y_{11}+y_{21}\right)+a_{1}x_{22}\left(y_{12}+y_{22}\right)=0, \end{array}$$

(35) $$\begin{array}{cc} \; & a_{0}x_{11}\left(y_{31}+iy_{41}\right)+a_{1}x_{12}\left(y_{32}+iy_{42}\right)\\ \; & \;\;\;\;\;\;-a_{0}x_{21}\left(y_{11}+iy_{21}\right)-a_{1}x_{22}\left(y_{12}+iy_{22}\right)=0, \end{array}$$

(36) $$\begin{array}{cc} \; & a_{0}x_{11}\left(y_{31}-y_{41}\right)+a_{1}x_{12}\left(y_{32}-y_{42}\right)\\ \; & \;\;\;\;\;\;+a_{0}x_{21}\left(y_{11}-y_{21}\right)+a_{1}x_{22}\left(y_{12}-y_{22}\right)=0, \end{array}$$

(37) $$\begin{array}{cc} \; & a_{0}x_{11}\left(y_{31}-iy_{41}\right)+a_{1}x_{12}\left(y_{32}-iy_{42}\right)\\ \; & \;\;\;\;\;\;-a_{0}x_{21}\left(y_{11}-iy_{21}\right)-a_{1}x_{22}\left(y_{12}-iy_{22}\right)=0. \end{array}$$

Similarly, we obtain:

(38) $$\begin{array}{ccc} a_{0}x_{11}y_{31}+a_{1}x_{12}y_{32}+a_{0}x_{21}y_{11}+a_{1}x_{22}y_{12} & = & 0, \end{array}$$

(39) $$\begin{array}{ccc} a_{0}x_{11}y_{41}+a_{1}x_{12}y_{42}+a_{0}x_{21}y_{21}+a_{1}x_{22}y_{22} & = & 0, \end{array}$$

(40) $$\begin{array}{ccc} a_{0}x_{11}y_{31}+a_{1}x_{12}y_{32}-a_{0}x_{21}y_{11}-a_{1}x_{22}y_{12} & = & 0, \end{array}$$

(41) $$\begin{array}{ccc} a_{0}x_{11}y_{41}+a_{1}x_{12}y_{42}-a_{0}x_{21}y_{21}-a_{1}x_{22}y_{22} & = & 0. \end{array}$$

Let us denote:

(42) $$M=\left(\begin{array}{cccc} a_{0}x_{11} & a_{1}x_{12} & a_{0}x_{21} & a_{1}x_{22}\\ a_{0}x_{21} & a_{1}x_{22} & a_{0}x_{11} & a_{1}x_{12}\\ a_{0}x_{11} & a_{1}x_{12} & -a_{0}x_{21} & -a_{1}x_{22}\\ a_{0}x_{21} & a_{1}x_{22} & -a_{0}x_{11} & -a_{1}x_{12} \end{array}\right)$$

and:

(43) $$y^{\left(1\right)}=\left(\begin{array}{c} y_{11}\\ y_{12}\\ y_{31}\\ y_{32} \end{array}\right),y^{\left(2\right)}=\left(\begin{array}{c} y_{21}\\ y_{22}\\ y_{41}\\ y_{42} \end{array}\right).$$

Then, the four equations (30), (32), (38), and (40) can be expressed as:

(44) $$My^{\left(1\right)}=0.$$

The other four equations (31), (33), (39), and (41) can be expressed as:

(45) $$My^{\left(2\right)}=0.$$

Based on the construction of the matrix *M* and *X* being a unitary matrix, we derived that *M* is invertible, and we obtain:

(46) $$y^{\left(1\right)}=y^{\left(2\right)}=0.$$

Thus, we know the determinant of matrix *Y* is equal to zero, which is a contradiction to *Y* being a unitary matrix. Therefore, we proved that the eight states (22), (23) form an unextendible maximally entangled basis.

## 6. Discussion and Conclusions

In this paper, we studied the construction of the MEBs in $C^{d}⊗C^{d^{\prime }}$. We provided a new construction of maximally entangled bases in $C^{d}⊗C^{d^{\prime }}$ when *d* is a divisor of $d^{\prime }$ and $d^{\prime }\geq 2d$. We also constructed unextendible maximally entangled bases in $C^{d}⊗C^{d^{\prime }}$ when $d^{\prime }=qd+r,q,r\in Z,q\geq 2,0<r<d$. We studied the examples of maximally entangled bases in $C^{2}⊗C^{6}$, $C^{3}⊗C^{6}$, $C^{2}⊗C^{4}$, and $C^{2}⊗C^{5}$, respectively. We found two pairs of maximally entangled bases in $C^{2}⊗C^{4}$, which are mutually unbiased bases. In $C^{2}⊗C^{5}$, we presented the eight-member unextendible maximally entangled basis. Our results will be helpful in studying the properties of MEBs. These results may be also helpful for further construction of unextendible bases and the research of quantum entanglement. We have not yet found a way to obtain more MEBs that are mutually unbiased bases based on our new construction. It would be interesting to investigate the relationships between the MUBs and the MEBs constructed by our methods in quantum entanglement and quantum computing.

## Data Availability

Not applicable.
